# The effect of transactional analysis on the self-esteem of imprisoned women: a clinical trial

**DOI:** 10.1186/s40359-019-0369-x

**Published:** 2020-01-13

**Authors:** Mahya Torkaman, Jamileh Farokhzadian, Sakineh Miri, Batool Pouraboili

**Affiliations:** 1 Student Research Committee, Department of Nursing, School of Nursing and Midwifery, Shiraz University of Medical Sciences , Shiraz, Iran; 2 Nursing Research Center, Kerman University of Medical Sciences, Kerman, Iran; 3 Department of Psychiatric Nursing, School of Nursing and Midwifery, Kerman University of Medical Sciences, Haft-Bagh Highway, PO Box: 7716913555, Kerman, Iran; 4 Department of pediatric and neonatal nursing, School of Nursing and Midwifery, Tehran University of Medical Sciences, Tehran, Iran

**Keywords:** Group training, Transactional analysis, Self-esteem, Imprisoned women

## Abstract

**Background:**

The imprisoned women usually have low self-esteem and suffer from various physical and mental complaints; they may suffer from feelings of emptiness, isolation, and depression. Transactional analysis (TA) is part of a comprehensive system attributed to the individual and social psychiatry for personal development of self-esteem among the imprisoned women. Therefore, the present study aimed to investigate the effect of TA group-training on the self-esteem of imprisoned women.

**Methods:**

This clinical trial was conducted among the imprisoned women in a prison in Southeastern Iran using pretest-posttest design. In this regard, 76 women were randomly allocated to the intervention (*n* = 35) and control (*n* = 41) groups. The TA group-training program was held for eight 90-min sessions for the intervention group. Data were collected using a demographic questionnaire and the Rosenberg’s self-esteem scale (RSES). Later, all participants were evaluated before and 1 month after the intervention.

**Results:**

In pre-test, the mean scores of self-esteem were 11.8 ± 4.67 and 7.97 ± 4.52 for the intervention and control groups, respectively. These scores showed low levels of self-esteem and the difference between the two groups was significant (*p* = 0.001, *t* = − 3.61). In the post-test, the mean scores of self-esteem improved to the moderate level (22 ± 2.52) in the intervention group compared to the control group (8.92 ± 4.04). This indicates the significant improvement of self-esteem in the intervention group (*p* = 0.001, *t* = 17.15).

**Conclusions:**

The results showed that TA group-training had a significant effect on self-esteem. Therefore, the experienced and expert counselors and psychologists are recommended to hold transactional analysis group-training courses to enhance self-esteem among women prisoners.

**Trial registration:**

Iranian Registry of Clinical Trials, IRCT20170725035289N5 Date registered: 25/08/2018.

## Background

Considering the rapid increase in the number of women prisoners, many studies have been conducted to understand their unique needs. The results of these studies showed that the pathway to prison differs between men and women [1].However, the low number of imprisoned women has led the authorities to neglect the problems and difficulties of these women; so, imprisoned women were put on the second rank of importance [2]. Women are often integral to families and the community health depends on them to a large degree, but many of them are faced with numerous unsolved problems and conflicts. Consequently, women’s misbehaviors and faults can threaten the stability and firmness of families [1]. Therefore, the self-confidence and mental aspect of the criminals, especially imprisoned women should be improved, so that they can restart a new and happy life after their imprisonment [3].

Self-esteem is a psychological concept that refers to an individual’s self-evaluation and indicates the extent to which people evaluate themselves as capable and worthy [4, 5]. In other words, self-esteem is defined as individuals’ subjective emotional response towards themselves [6]. Psychologists usually define self-esteem as an enduring personality characteristic with possibly normal short-term variations. Self-esteem is a concept of personality [7]. In order to develop self-esteem, a sense of self-worth is required, which is achieved by embracing challenges that result in success [8].

High or low self-esteem causes numerous positive and negative effects on humans. Individuals with high self-esteem are always dutiful and responsible; such characteristics are created as the consequence of awareness about one’s actions and behaviors [9]. On the other hand, individuals with low self-esteem ignore their goals much more easily and move through the direction determined by others [7]. Self-esteem is an important factor for the individual’s progress and success throughout life. Individuals with positive evaluation of their characteristics have different great goals. They are also empowered with more enthusiasm and eagerness to face new successes [10]. Therefore, individuals’ “self-interpretation and ego state” can be considered as an effective factor in their self-esteem. In this regard, TA is introduced as a useful therapeutic approach for individuals with low self-esteem [11].

Eric Berne, the founder of TA, indicated that individuals manifest certain sets of thoughts, feelings, and behaviors at different situations [12]. Berne called these three sets as “Ego States” of Child, Adult, and Parent. Due to the fact that each state of Ego is considered as one of the sub-constructs of ego, each state would be adjusted and adapted if used in an appropriate situation [13]. Each Ego-state is characterized by a particular set of beliefs, emotions, and behavior responsible for social interactions [11]. The Ego-Child state is characterized by impulsivity, emotionality, expressivity, irrational behavior, and a self-centered attitude. The Ego-Parent state is characterized by a set of values, norms, orders, prohibitions, and obligations [14]. The Ego-Adult state is characterized by mostly logical constructive conditioning. This state is responsible for the reality analysis, estimation of the possible solutions, rational decision makings, and assertive relationship of the individual with others [15]. The literature showed that TA group-training method was an effective method in helping people to communicate and interact with other individuals [16]. In general, TA has been used to increase self-esteem [11], help anxious and abused women [17], and treat personality disorders [14]. Furthermore, it has been widely applied in clinical and therapeutic affairs [15], developmental psychology [18], and management communication of personality, behaviors, and relationships [15, 16, 19]. Although TA is one of the most effective psychological theories, it has not been considered much by the researchers [18]. Techniques of TA were designed to promote the independence and self-direction using one’s knowledge, self-motivation, and resources as an adult to solve problems [16, 20]. The effectiveness of TA training on self-esteem was confirmed in some studies [15]. In Yugoslavia, a study over the alcohol and non-alcohol addicts indicated that the TA group-training had a positive effect on the psycho-emotional state of the alcohol addicts [14]. A research in Iran showed that addicted women had a low level of self-esteem, which hindered them from changes and reforms in life. The findings of the mentioned study recommended TA to improve self-esteem of the addicted imprisoned women [6]. Another study in Iran was carried out by Forghani et al. They reported that TA had a significant impact on the addicts and prevented addiction recurrence by enhancing the participants’ coping skills and mental functions [21].

The studies mentioned above were conducted to investigate the effect of TA on the self-esteem or other psychological characteristics of individuals. Women, as the main members of the society and family, have been neglected most of the time. To the best of our knowledge, the effect of TA group-training on the self-esteem of Iranian imprisoned women has not been investigated. Therefore, the present study was conducted to deal with the issue.

## Methods

### Study design & setting

This pretest-posttest randomized clinical trial was conducted in the Southeast of Iran. In the prisons of Iran, monitored by the prison-related organizations of each province, different specialists work, such as psychologists and psychiatrists to help the prisoners. Sports classes are also held by the authorities. According to the rules, imprisoned women can meet their families on certain days of week and in some crimes, they can have some leaves per in the month.

### Samples

The target population of this study included al the imprisoned women at the time of data collection (*N* = 120). The study participants were 84 imprisoned women selected according to the inclusion criteria. The participants were randomly allocated to the intervention and control groups (42 participants in each group). The inclusion criteria of this study recruited the prisoners who were in prison for at least 6 months, whose jail sentences were up to the end of study, and who had at least elementary education. The exclusion criteria consisted of failure to complete the questionnaires due to any reasons and failure to attend the training sessions for more than one session. In total, 35 questionnaires in the intervention and 41 questionnaires in the control groups were collected and analyzed.

### Instrument and data collection

Data were collected using two questionnaires. The first one was a demographic questionnaire and the second one was the 10-item Rosenberg Self-Esteem Scale (RSES) developed in 1965. This scale evaluates the individual’s overall self-image. Each item in RSES is scored based on a four-point Likert scale including ‘totally agree’ (3 scores), ‘agree’ (2 scores), ‘disagree’ (1 score), and ‘totally disagree’ (0 score). The negative items (items from 6 to 10) were scored inversely. In RSES, higher scores indicate higher self-esteem levels and the attainable scores range from zero to 30. Additionally, low, moderate, and high levels of self-esteem are indicated by scores lower than 15, 15–25, and higher than 25, respectively. The Cronbach’s alpha for the original scale was measured as 74% [22, 23] (Additional file 1). In a study by Rajabi et al., the internal consistency coefficient of the Persian version of the scale was calculated as 84% for the entire sample of students, 87% for the male students, and 80% for the female students. The correlation coefficients between the score of each item and the total score varied from 56 to 72%, which were significant at *P* < 0.001. The construct validity of the scale was measured using factor analysis. Moreover, a negative and significant relationship was observed between this scale and the Death Obsession Scale (DOS) with a coefficient of − 0.34 for the entire sample, − 0.44 for the male students, and − 0.27 for the female students. This suggests the good validity of the Rosenberg’s Self-Esteem Scale [24].

### Intervention procedure

Initially, the experimental group was divided into three sub-groups (14 participants in each). Then, sub-each group attended the TA training program in eight 90-min sessions weekly. The TA trainings were presented by the first researcher, a psychologist, a nursing PhD, and a psychiatric nurse using educational slides, lectures, group discussions, as well as questions and answers. The control group received no training during the study period. Table 1 represents the topics presented during the training sessions [25].

**Table 1:** Training program

Session 1:	Establishing initial communication, Introducing members to each other, and clarifying the study purpose
Session 2:	Introducing the initial concepts of the structural analysis: “parent”, “adult “, and “child”.
Session 3:	Getting acquainted with the concept of mutual communication: direct transaction, crossed transaction, and ulterior transaction.
Session 4:	Strengthening the “adult” and controling the negative aspects of the “parent”, especially the “critical parent”.
Session 5:	Explaining about the personality illnesses, providing various examples, and considering the interpersonal exchanges.
Session 6:	Explaining time management using different methods.
Session 7:	Life minute analysis: Discussing the life formation process, omitting the undesirable events in life, and making decisions applying the “Adult”.
Session 8:	Making healthy relationships with others and adjustments to different situations. Increasing intimacy, adopting a healthy state of life, and having conscious control of the Ego states. Discussing and making conclusions about the subject.

### Statistical analysis

Data were analyzed using SPSS (version 20) and by running descriptive (percentage, mean, and standard deviation) and analytical statistics (chi-squared test, independent samples *t*-test, and paired-samples *t*-test and ANCOVA). According to the results of Kolmogorov-Smirnov test, data enjoyed a normal distribution. The level of significance was set at *P* ≤ 0.05.

## Results

### Demographic information

The results showed that the majority of participants were housewives in the age range of 21–40 years and experienced their first prison sentence. Most participants had elementary education, were married, and were prisoned for 3 years. According to the chi-squared test, no significant difference was observed between the intervention and control groups in terms of the demographic information (Table 2).

**Table 2:** Comparison of the demographic information between the intervention and control groups

Variables	Groups	Intervention		Control		*χ2*	*P*
		n	٪	N	٪		
Occupation	Housekeeper	35	100	41	92.70	2.66	0.446
	Self-employed	0	0	1	2.40		
	Clerk	0	0	1	2.40		
	Others	0	0	1	2.40		
Age	≤20	1	2.90	1	2.40	0.336	0.953
	21–40	24	68.60	26	63.40		
	41–60	9	25.70	13	31.70		
	> 60	1	2.90	1	2.40		
History of imprisonment	Once	15	42.90	18	43.90	0.009	0.995
	Twice	14	40	16	39		
	≥Three times	6	17.10	7	17.10		
Place of residence	Native	34	97.10	41	100	1.18	0.276
	non- Native	1	2.90	0	0		
Education	Primary School	2	5.70	0	0	4.95	0.175
	High School	23	65.70	30	56.60		
	Diploma	8	22.90	11	26.80		
	University Education	2	5.70	0	0		
Marital status	Single	1	2.90	0	0	2.51	0.473
	Married	27	77.10	34	82.90		
	Divorced	4	11.40	2	4.90		
	Widow	3	8.60	5	12.20		
Prison term)Year)	1–2 Years	12	34.30	13	31.70	0.570	0.812
	≥3	23	65.70	28	68.30		

### Self esteem

In the pre-test stage, the mean score of self-esteem in the intervention group (11.8 ± 4.67) was higher than that of the control group (7.97 ± 4.52) and the difference between the two groups was significant (*p* = 0.001, *t* = −3.61). However, the results showed that the level of self-esteem was low for both groups. In the post-test stage, the mean score of self-esteem in the intervention group (22 ± 2.52) was significantly higher than that of the control group (8.92 ± 4.04) (*p* = 0.001, *t =* 17.15). In other words, the intervention group members were at the low level of self-esteem prior to the intervention, but after attending the educational classes, their self-esteem increased into the moderate level, which indicates the significant effect of the intervention (Table 3).

**Table 3:** The pretest and posttest scores of the control and intervention groups

Groups	Pre test	Post test	Statistic t^a^& p
	M ± SD	M ± SD	
Intervention	11.8 ± 4.67	22.00 ± 2.52	*t* = − 12.15
			*P =* 0.001
Control	7.97 ± 4.52	8.92 ± 4.04	*t* = − 2.79
			*P* = 0.008
Statistic *t*^b^ *& p*	*t* = 3.61	*t* = 17.5	
	*P* = 0.001	*P* = 0.001	

^a^Paired-t-test, ^b^independent-t-test

The covariance analysis test to control the impact of pretest on the self-esteem of women prisoners are presented in Table 4. These results confirmed the results in Table 3.

**Table 4:** Summary of covariance analysis for the control and intervention groups

Variable	Type II sum of square	Df	Mean square	F	*p*-value
Corrected model	3543.22	2	1771.66	233.20	< 0.001
Intercept	1600.57	1	1600.57	210.69	< 0.001
Pre-test	316.21	1	316.21	41.62	< 0.001
Group	2068.05	1	2068.05	272.22	< 0.001
Error	554.56	73	7.59		< 0.001

## Discussion

The purpose of this study was to investigate the effect of TA group-training on the self-esteem of imprisoned women. The results showed that the majority of female prisoners were at low levels of self-esteem before implementing the group-training program, which is consistent with the findings of many studies.

For example, a study among the addicted female prisoners in South of Iran represented that most prisoners had low self-esteem [6]. Arefi reported that female prisoners, especially those with commission crimes, had low self-esteem [26].The similarity of these results with those of the present study can be due to the same research population and data collection tools.

Furthermore, a reason for the low level of self-esteem among the imprisoned women can be attributed to the prison conditions and environment. In addition, the prisoners’ isolation and being away from their families, fear of rejection in society, and lack of social protection during and after their sentence are among some other effective factors. Alavi showed that Iranian female prisoners had a moderate level of self-esteem. Alavi also introduced low self-esteem as a predictive factor of drug abuse, robbery, and prostitution [27]. Moreover, Kamoyo reported that self-esteem was at a moderate level among the Kenyan female prisoners [28]. The discrepancy between results of the above-mentioned studies and the present study can be probably due to the different cultures of various societies in accepting women, especially female prisoners as well as the different data collection tools.

Results of our study indicated that TA group-training significantly improved the self-esteem of intervention group from the “low” level to the “moderate” Level. Unfortunately, no published study has ever examined the effect of TA group-training as a strategy to improve self-esteem and psychological well-being of the imprisoned women with mental health issues.

Other studies reported TA as an effective reconsideration method in life, which helps the individuals to feel valuable and solve their problems successfully [16, 17, 29]. Another study on the impact of TA group-training indicated improvement of the self-confidence and the ability to endure failures [18] among members of the intervention group. In several studies, participants reported more success in family life and social relationships [19], reduced mental and psychological stresses [30], increased self-esteem, better control of personal conflicts, and more decisiveness in achieving the goals and ideals [31].The results of a study by Riaz et al. showed that cognitive behavioral therapy (CBT) group programs increased the prisoners’ self-acceptance and self-admission and reduced their risky behaviors [32].Furthermore, Khodayarifard et al. conducted the individual and group (combined) cognitive–behavioral interventions and showed improvement in the psychological well-being of the prisoners. Finally, they concluded that these strategies increased the female prisoners’ self-efficacy and self-esteem [3].Perhaps, the similarity between the results of these studies can be due to similar intervention methodology and participation in the group discussion. In group therapy sessions, participants are exposed to other participants’ experiences and contributions. Therefore, they start to analyze and scrutinize their own problems. In addition, the inmates can benefit from other individuals’ opinions and discuss about their problems.

We also found that the imprisoned women in Iran were highly aware of their own low self-esteem and had a tendency to express it through avoidance behaviors. These participants used more emotion-focused than problem-focused coping strategies to overcome their low self-esteem (e.g., active coping, planning, and instrumental support).

Considering that female prisoners in Iran suffer from low self-esteem, which leads into their avoidance behaviors, special group programs, such as TA trainings should be conducted for them by specialties. Consequently, the female prisoners can adapt themselves to the conditions in the community and return to their families by self-acceptance and self-admission.

Our study had a number of limitations. First, the imprisoned women had short-term leaves, family visits, communication with counselors, clinical psychologists, and social workers in prison. These factors could affect the results, but were out-of-control. Second, we could not find a precise instrument to assess the self-esteem of the imprisoned women, therefore, RSES was used. Third, the researchers had access to the prisoners for only 3 months and could conduct only one follow-up 1 month after theintervention. One month is a very short period to assess the impact of TA on a complex behavior such as self-estem. Fourth, the therapist was not permitted to conduct the therapeutic sessions in a separate room. The only available space was a room next to the jailer’s office. Furthermore, some security staff attended the ongoing sessions. These factors disturbed the privacy of the intervention group. Fifth, the results might have changed in the case that the control group received some kinds of training, such as strengthening the back muscles or excersizing. Sixth, since the differnce between the two groups was significant at the pre-test with regard to the self-esteem scores, future researchers are recommneded to select participants based on their pre-test mean scores of self-esteem, instead of a random allocation to the intervention and control groups. In this way, both groups would have women with comparable mean scores.

Therefore, further research are suggested using a valid and reliable tool to assess the self-esteem of the imprisoned women. Moreover, longitudinal studies should be conducted bycontroling the confounding variables.

## Conclusion

Results of the present study showed that TA had a significantly positive effect on the imprisoned women in the intervention group. Therefore, prison managers are recommended to conduct TA group-trainings with the help of psychologists and psychiatric nurses to strengthen self-esteem of the imprisoned women.

Rehabilitative programmers are also suggested to employ the self-esteem strategy in prisons to improve the prisoners’ self-esteem. Furthermore, the prison environment should be modified to allow for more frequent presence of social support groups. In this regard, we recommend other researchers to carry out quantitative and qualitative studies on the effective strategies to overcome the barriers against self-esteem among female prisoners. Future studies can also examine the effect of TA group-training on other psychological aspects of the imprisoned women in various cultures and contexts.

## Supplementary information

**Additional file 1.** Rosenberg Self-Esteem Scale

## Data Availability

The datasets generated and analysed in the present study, are available upon request to the corresponding author after signing appropriate documents in line with ethical application and the decision of the Ethics Committee.

## Abbreviations

RSES: Rosenberg’s self-esteem scale
TA: Transactional analysis
