# Correction to: The effect of transactional analysis on the self-esteem of imprisoned women: a clinical trial

**DOI:** 10.1186/s40359-020-0382-0

**Published:** 2020-02-06

**Authors:** Mahya Torkaman, Jamileh Farokhzadian, Sakineh Miri, Batool Pouraboli

**Affiliations:** 1 Student Research Committee, Department of Nursing, School of Nursing and Midwifery, Shiraz University of Medical Sciences, Shiraz, Iran; 2 Nursing Research Center, Kerman University of Medical Sciences, Kerman, Iran; 3 Department of Psychiatric Nursing, School of Nursing and Midwifery, Kerman University of Medical Sciences, Haft-Bagh Highway, PO Box: 7716913555, Kerman, Iran; 4 Department of pediatric and neonatal nursing, School of Nursing and Midwifery, Tehran University of Medical Sciences, Tehran, Iran

**Correction to: BMC Psychol**

**https://doi.org/10.1186/s40359-019-0369-x**

Following publication of the original article [1], the authors advised that the name of the 4th author had been submitted incorrectly; the author has the family name ‘Pouraboli’, however, their article was originally published with the family name (mis) spelled as ‘Pouraboili’.

Please find the corrected name in the author list of this article.

The error has now been corrected in the original article.

The authors apologize for any inconvenience caused.
