# Supplementary material for: The effect of transactional analysis on the self-esteem of imprisoned women: a clinical trial
Source: BMC Psychol. 2020 Jan 13;8:3. doi: 10.1186/s40359-019-0369-x (PMC6958781; doi:10.1186/s40359-019-0369-x)
Supplement: Supplementary file 1 — Additional file 1. Rosenberg Self-Esteem Scale [file 40359_2019_369_MOESM1_ESM.docx]

**Rosenberg Self-Esteem Scale**

Below is a list of statements dealing with your general feelings about yourself. Please indicate how strongly you agree or disagree with each statement.

| **STATEMENT** | **Strongly Agree** | **Agree** | **Disagree** | **Strongly Disagree** |
| --- | --- | --- | --- | --- |
| 1.On the whole, I am satisfied with myself. |  |  |  |  |
| 2.At times I think I am no good at all. |  |  |  |  |
| 3.I feel that I have a number of good qualities. |  |  |  |  |
| 4.I am able to do things as well as most other people. |  |  |  |  |
| 5.I feel I do not have much to be proud of. |  |  |  |  |
| 6.I certainly feel useless at times. |  |  |  |  |
| 7.I feel that I'm a person of worth, at least on an equal plane with others. |  |  |  |  |
| 8.I wish I could have more respect for myself. |  |  |  |  |
| 9.All in all, I am inclined to feel that I am a failure. |  |  |  |  |
| 10.I take a positive attitude toward myself. |  |  |  |  |
